# Exposure to air pollution and cognitive impairment risk: a meta-analysis of longitudinal cohort studies with dose-response analysis

**DOI:** 10.7189/jogh.10.010417

**Published:** 2020-06

**Authors:** Xiaohui Yu, Liwen Zheng, Wenjie Jiang, Dongfeng Zhang

**Affiliations:** Department of Epidemiology and Health Statistics, the School of Public Health of Qingdao University, People’s Republic of China

## Abstract

**Background:**

We conducted a meta-analysis to explore the relationship between exposure to air pollution and the risk of cognitive impairment of longitudinal cohort studies.

**Methods:**

PubMed, Web of Science and Wan Fang databases were searched for relevant articles of longitudinal cohort studies published between January 1950 and September 2019. The pooled relative ratio (RR) and 95% confidence interval (CI) were calculated using the random effect model.

**Results:**

Ten articles involving 519 247 cases among 12 523 553 participants were included in this meta-analysis. The pooled RR of cognitive impairment per 5 μg/m^3^ increments in exposure to particulate matter ≤2.5 μm (PM_2.5_) was 1.08 (95% CI = 1.03, 1.13; *I^2^* = 82.2%; *P_heterogeneity_*<0.001). No association was found between nitrogen dioxide/nitrogen oxide (NO_2_/NO_x_) and ozone (O_3_) and cognitive impairment. For PM_2.5_ exposure, in subgroup analysis, the above-mentioned significant positive association was found among studies conducted in population (RR*_p_*_er_ *_5 μg/m_^3^ =* 1.05; 95% CI = 1.01,1.09; *I^2^* = 57.4%; *P_heterogeneity_* = 0.016), in North America (RR*_per 5 μg/m_^3^* = 1.13; 95% CI = 1.01,1.26; *I^2^* = 86.7%; *P_heterogeneity_*<0.001) and with follow-up duration >10 years (RR*_p_*_er_ *_5 μg/m_^3^* = 1.10; 95% CI = 1.03,1.17; *I^2^* = 86.3%; *P_heterogeneity_*<0.001).

**Conclusions:**

This meta-analysis suggests that exposure to PM_2.5_ might increase the risk of cognitive impairment.

Air pollution, mainly particulate matter (PM) and gas pollutants [1], is one of the ten threats to global health in 2019, causing 7 million people dying prematurely every year [2]. Updated estimations from the World Health Organization (WHO) show that around 90% of the worldwide people are breathing polluted air [3]. Hence, air pollution has become the greatest environmental hazard to public health.

Cognitive impairment, mainly encompassing both Alzheimer disease (AD) and dementia [4-6], is the 5^th^ leading cause of global death in 2016 [7], occurring nearly 10 million new cases every year [8]. The cause of cognitive impairment is considered to be a combination of genetic and environmental factors. As a modifiable environmental factor, air pollution plays a crucial role in central nervous system diseases, including cognitive impairment [9]. Accumulating animal studies indicated that air pollutants could lead to neuroinflammation and oxidative stress [9-11], which were involved in pathological evidence of cognitive impairment [12-16]. Based on these findings, many studies have evaluated the association between air pollution and cognitive impairment. Specifically, for PM_2.5_ exposure, a positive association was found in four studies [17-19], whereas no significant association was shown in other studies [20-25]. For NO_2_/NO_x_ exposure, although the risk of cognitive impairment was illustrated in two studies [17,18], the link was not manifested in other studies [20,21,25,26]. For O_3_ exposure, one study [17] revealed an inverse relationship with cognitive impairment, while another study [20] found a positive relationship with cognitive impairment. In light of the inconsistencies among the above epidemiological studies, we conducted a meta-analysis of longitudinal cohort studies to synthesize the results of existing studies to evaluate the relationship of air pollution with cognitive impairment.

## METHODS

This meta-analysis was conducted according to the Preferred Reporting Items for Systematic reviews and Meta-Analyses (PRISMA) guidelines [27].

### Search strategy

We searched all relevant articles in English or Chinese from PubMed, Web of Science and Wan Fang databases published between January 1950 and September 2019. The following search strategy was used: (air pollution or particulate matter or carbon monoxide or nitrogen dioxide or nitrogen oxide or sulfur dioxide or ozone) And (cognitive impairment or dementia). The exhaustive search process in PubMed is shown in Table S1 in the **Online Supplementary Document**. Furthermore, we reviewed the reference lists of retrieved articles to identify additional relevant articles.

### Inclusion criteria

The included studies ought to meet the following criteria: (1) longitudinal cohort studies published in English or Chinese; (2) the exposure of interest was particulate (PM_2.5_, particles with an aerodynamic diameter ≤2.5 μm; PM_10_, particles with an aerodynamic diameter ≤10 μm or PM_2.5-10_, particles with an aerodynamic diameter 2.5-10) or gaseous (NO_2_, nitrogen dioxide; NO_x_, nitrogen oxide; O_3_, Ozone; CO, carbon monoxide; SO_2_, sulfur dioxide) air pollutants; (3) the outcome of interest was cognitive impairment mainly including dementia and AD; (4) Multivariate adjusted odds ratio (OR), relative risk (RR) or hazard ratio (HR) and their 95% confidence interval (*CI*) of cognitive impairment with exposures to air pollution with per unit increase in pollutant concentrations (μg/m^3^, mg/m^3^, ppb, or ppm) were available, or sufficient data could be used to convert these results.

### Exclusion criteria

(1) Review articles and studies were written in other languages instead of English or Chinese; (2) studies that did not estimate the relationship between air pollution and the risk of cognitive impairment; (3) results cannot be converted into RR and 95% CI with per unit increase in particulate (PM_2.5_, PM_10_, or PM_2.5-10_) or gaseous (NO_2_, NO_x_, O_3_, CO, SO_2_) air pollutants concentrations (μg/m^3^, mg/m^3^, ppb, or ppm); (4) duplicated articles; (5) not cohort studies.

Two investigators independently performed the literature search. If there was disagreement on an article, the consensus was reached through discussion. If data appeared in more than one study, we would select the most comprehensive data.

### Data extraction

Data extracted from each identified study by two investigators independently as follows: the first author’s name, publication year, study areas and country, age range or mean age of participants, sex, follow-up duration, sample size, number of cases, measurement of exposure, the source of outcome, RR*s* (we presented all results with RR for simplicity) and their 95% CI with per unit increase in particulate (PM_2.5_, PM_10_, or PM_2.5-10_) or gaseous (NO_2_, NO_x_, O_3_, CO, SO_2_) air pollutants concentrations (μg/m^3^, mg/m^3^, ppb, or ppm), adjusted covariates.

The included cohort literature was assessed by the Newcastle-Ottawa Scale (NOS) [28].

### Statistical analysis

We use the inverse variance and the number of participants to weight the study-specific log RR*s* that can calculate the pooled RR with corresponding 95% CI to evaluate the relationship between air pollution and cognitive impairment. For consistency, all RR*s* were standardized to an increment of 5 μg/m^3^ of particular matter (PM_2.5_, PM_2.5-10_, and PM_10_) concentration and 5 ppb of gaseous (NO_2_, NO_x_, O_3_, CO, SO_2_) concentration. It is hypothesized that there is a linear relationship of exposure to air pollution and cognitive impairment. If studies reported RR*_u_* per u units not in per 5 μg/m^3^ of particular matter (PM_2.5_, PM_2.5-10_, and PM_10_) or 5 ppb of gaseous (NO_2_, NO_x_, O_3_, CO, SO_2_), the RR*_standardized_* was computed by the following formula [29]:

RR*_standardized_* = RR*_u_*^increment unit (eg, 5)/u^

Where u represents the increment utilized in the original study to assess the effects. We used a unit conversion factor: 1 ppb = 1.88 μg/m^3^ for NO_2_/NO_x_ and 1 ppb = 1.96 μg/m^3^ for O_3_, and standard condition is ambient pressure of 1 atmosphere and a temperature of 25°C [30].

We adopted to *I^2^* statistic to evaluate the heterogeneity, and the *I^2^* values of 0%, 25%, 50%, and 75% represent no, low, moderate and high heterogeneity, respectively [31]. The random effect model (REM) was utilized as the pooling method. Meta-regression with restricted maximum likelihood estimation was used to explore the potential covariates that may exert substantial impacts on between-study heterogeneity [32]. Subgroup analysis was conducted by the source of outcome, study areas, follow-up duration, and adjustment status for comorbidity and smoking. We carried out the leave-one-out sensitivity analysis to examine whether individual study influences between-study heterogeneity [33]. Influence analysis was performed excluding one study at a time to ascertain whether the aggregate results could be significantly affected by a single study [34]. Additionally, the funnel plot and Egger regression asymmetry test were used to estimate the publication bias [35]. All statistical analyses were done using STATA Version 15.0 (STATA Corporation, College Station, TX, USA). A two-sided *P* ≤ 0.05 was equated statistically significant.

## RESULTS

### Search results and study characteristics

**Figure 1:**
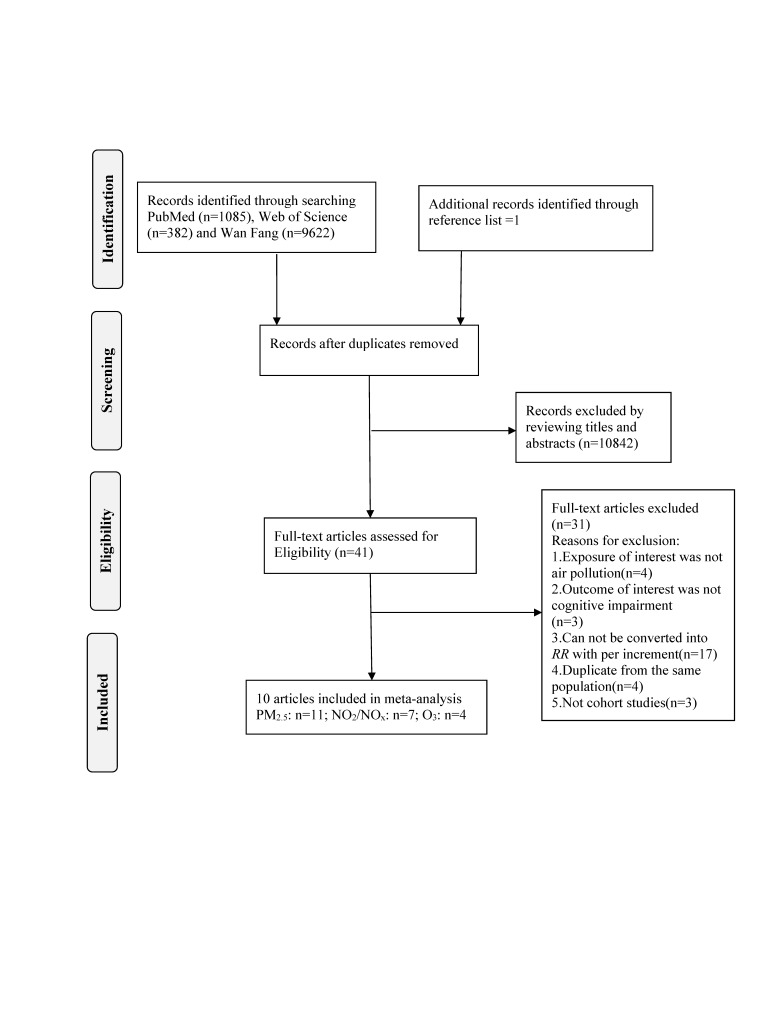
Flow diagram of the literature search for the study selection process.

On the basis of search strategy, a total of 11 090 articles were identified, involving 1085 articles from PubMed, 382 articles from Web of Science, 9622 articles from Wan Fang and one article from reference lists. After removing 207 articles because of duplicates, there are 10 883 articles left. Reviewing the title and abstract later, forty-one articles were remaining. We further excluded thirty-one articles after reviewing the full-text. The flow diagram of the literature search is displayed in **Figure 1**. The detailed reason for full-text reviewed articles exclusion is provided in Table S2 in the **Online Supplementary Document**.

Ultimately, ten articles [17-26], including 11 studies on PM_2.5_, 7 studies on NO_2_/NO_x_ and 4 studies on O_3_, were satisfied the inclusion criteria and were enrolled in this meta-analysis. Eight articles [18,21-26] were population-based studies and two articles [19,20] were hospital-based studies. The duration of follow-up ranged from 4 to 18 years, with three articles [17,22,23] following equal to or less than 10 years and seven articles [18-21,24-26] more than 10 years. Concerning the study areas, four articles [17,20,24,26] were performed in Europe, five articles [18,19,21,23,25] in North America and one article [22] in Asia.

The quality assessment indicated that the Newcastle-Ottawa score of cohort studies was not less than 7. Specific quality assessments are presented in Table S3 in the **Online Supplementary Document**. The characteristics of the studies are shown in **Table 1**.

**Table 1:** Characteristics of included studies on the association between air pollution and the risk of cognitive impairment*

Author, (year) [Ref.]	Country	Age (year), Sex	Follow-up years	Sample size (cases)	Exposure	Exposure measurement	Outcome	Source of outcome	RR (95% CI)	Adjustments
Carey (2018) [17]	Britain	50-79, Both	9	130 978 (2181)	PM_2.5_, NO_2_, O_3_	Dispersion mode	Dementia	Population based	PM_2.5_, original value (Per IQR = 0.95μg/m^3^): 1.06 (1.02, 1.12); equivalent value (Per 5 μg/m^3^): 1.36 (1.11, 1.82).	Age, sex, ethnicity, smoking, body mass index, index of multiple deprivation comorbidity (ischemic heart disease, stroke, heart failure).
									PM_2.5_ (traffic), original value (Per IQR = 0.58μg/m^3^): 1.08 (1.01, 1.16); equivalent value (Per 5 μg/m^3^): 1.94 (1.09, 3.59).	
									NO_2_, original value (Per IQR = 7.47μg/m^3^): 1.16 (1.05, 1.27); equivalent value (Per 5 ppb): 1.21 (1.06, 1.35).	
									O_3_, original value (Per IQR = 5.56μg/m^3^): 0.85 (0.76, 0.94); equivalent value (Per 5 ppb): 0.75 (0.62, 0.90).	
Oudin (2016) [26]	Sweden	55-85, Both	18	1806 (302)	NO_x_	LUR	Dementia	Population based	Original value (Per 10 μg/m^3^): 1.05 (0.98, 1.12); equivalent value (Per 5 ppb): 1.05 (0.98, 1.11).	Baseline age, sex, education, physical activity, smoking, body mass index, waist–hip ratio, alcohol, ApoE4, baseline medical history of diabetes, stroke and hypertension.
Oudin (2018) [24]	Sweden	55-85, Both	18	1806 (302)	PM_2.5_	Dispersion mode	Dementia	Population based	Wood burning, original value (Per 1μg/m^3^): 1.55 (1.00, 2.41); equivalent value (Per 5 μg/m^3^): 8.95 (1.00, 81.30).	Age, sex, PM_2.5_ from traffic exhaust, PM_2.5_ from residential wood burning, physical activity, smoking, body mass index, alcohol and waist-hip-ratio.
									Traffic exhaust, original value (Per 1μg/m^3^): 1.14 (0.59, 2.23); equivalent value (Per 5 μg/m^3^): 1.93 (0.07, 55.15).	
Chen (2017) [18]	Canada	55-85, Both	13	2 066 639 (257 816)	PM_2.5_, NO_2_, O_3_	LUR	Dementia	Population based	PM_2.5_, original value (Per IQR = 4.8μg/m^3^): 1.04 (1.03, 1.05); equivalent value (Per 5 μg/m^3^): 1.04 (1.03, 1.05).	Age, sex, history of diabetes, hypertension, coronary heart disease, stroke, congestive heart failure, arrhythmia, traumatic brain injury, income quintile, urban residency, north/south indicator, census division-level unemployment rate, education and recent immigrants.
									NO_2_, original value (Per IQR = 14.2 ppb): 1.10 (1.08, 1.12); equivalent value (Per 5 ppb): 1.034 (1.027,1.041).	
									O_3_, original value (Per IQR = 6.3 ppb): 0.98 (0.96, 1.00); equivalent value (Per 5 ppb): 0.98 (0.97, 1.00).	
Cerza (2019) [20]	Italy	65-100, Both	13	350 844 (21 548)	PM_2.5_, NO_2_, NO_x_ O_3_	LUR and chemical transport model	Dementia	Hospital based	PM_2.5_, original value (Per 5 μg/m^3^): 0.99 (0.96, 1.02).	Age, education, place of birth, marital status, area-based socioeconomic position with baseline hazard function stratified by sex.
									NO_2_, original value (Per 10 μg/m^3^): 0.97 (0.96, 0.99); equivalent value (Per 5 ppb): 0.97 (0.96, 0.99).	
									NO_x_, original value (Per 20 μg/m^3^): 1.01 (1.00, 1.02); equivalent value (Per 5 ppb): 1.005 (1.000, 1.009).	
									O_3_, original value (Per 10 μg/m^3^):1.06 (1.03, 1.08); equivalent value (Per 5 ppb): 1.06 (1.03, 1.08).	
Kioumourtzoglou (2016) [19]	America	75, Both	12	9 817 806 (230 463)	PM_2.5_	The US Environmental Protection Agency’s (EPA) Air Quality System (AQS) database	Dementia	Hospital based	Original value (Per 5 μg/m^3^): 1.46 (1.29, 1.66).	Age, sex, race, individual socioeconomic status and for any prior cardiopulmonary admission and severity of disease.
Ilango (2019) [21]	Canada	60, Both	18	34 391 (2559)	PM_2.5_, NO_2_	LUR	Dementia	Population based	PM_2.5_, original value (Per 10 μg/m^3^): 1.29 (0.99, 1.64); equivalent value (Per 5 μg/m^3^): 1.14 (0.99, 1.28).	Age, sex, education, marital status, income quintile, smoking status, body mass index, physical activity, rural residence and northern region; area level: recent immigrants, unemployment and education.
									NO_2_, original value (Per 5 ppb): 1.10 (0.99, 1.19).	
Yu (2016) [25]	Canada	≥65, Both	14	5249 (1346)	PM_2.5,_ NO_2_	Satellite imaging and LUR	Dementia	Population based	PM_2.5_, original value (Per 10 μg/m^3^): 1.14 (0.93, 1.40); equivalent value (Per 5 μg/m^3^): 1.07 (0.96, 1.18).	Age, sex, vitamin use, non-steroidal anti-inflammatory drugs, exercise, hypertension, and obesity.
									NO_2_, original value (Per 5 ppb): 0.98 (0.93, 1.03).	
Jung (2015) [22]	China	≥65, Both	10	95 690 (1399)	PM_2.5_, O_3_	Monitoring stations	Alzheimer disease	Population based	PM_2.5_, original value (Per 13.21μg/m^3^): 1.03 (0.95, 1.11); equivalent value (Per 5 μg/m^3^): 1.01 (0.98, 1.04).	Age, sex, income, diabetes mellitus, hypertensive disease, myocardial infarction, stroke, asthma and chronic obstructive pulmonary disease.
									O_3_, original value (Per 9.63 ppb): 1.06 (1.00, 1.12); equivalent value (Per 5 ppb): 1.03 (1.00,1.06).	
Loop (2013) [23]	America	64, Men: 45%	4	20 150 (1633)	PM_2.5_	Ground-level and Earth-orbiting satellite measurements	Cognitive impairment	Population based	Original value (Per 10 μg/m^3^): 0.98 (0.72, 1.34); equivalent value (Per 5 μg/m^3^): 0.99 (0.85, 1.16).	Age, race, region, education, body mass index, income, PM_2.5_, length of follow up, the potential confounders temperature,season,incident stroke, smoking status, alcohol use, exercis and comorbidities (presence of depressive symptoms, diabetes,dyslipidemia, hypertension).

RR – relative risk, PM_2.5_ – particulate matter ≤2.5 μm, NO_2_ – nitrogen dioxide NO_x_ – nitrogen oxide, O_3_ – ozone; LUR – land-use regression model; Equivalent value, RR _standardized_ = RR_u_^increment unit (eg, 5)/u^ is the equation for converting the original value to the equivalent value. RR _standardized_ denotes equivalent value, RR_u_ denotes original value, u denotes the increment of PM_2.5_, NO_2,_ NO_x,_ O_3_ concentration used in the original study, ppb - parts per billion

*Conversion factors: NO_2_/NO_x_: 1 ppb = 1.88 μg/m^3^; O_3_, 1 ppb = 1.96 μg/m^3^.

### Quantitative synthesis

**Table 2** shows the summary risk estimates of cognitive impairment for air pollution according to study characteristics.

**Table 2:** Summary risk estimates of cognitive impairment for air pollution according to study characteristics

Study characteristics	PM_2.5_	RR (95% CI)	*I^2^* (%)	*P_heterogeneity_*	NO_2_/NO_x_	RR (95% CI)	*I^2^* (%)	*P_heterogeneity_*
N	N
All studies	11	1.08 (1.03, 1.13)	82.2%	<0.001	7	1.02 (0.99, 1.04)	93.4%	<0.001
Source of outcome:								
Population-based	9	1.05 (1.01, 1.09)	57.4%	0.016	5	1.048 (1.004, 1.094)	68.5%	0.013
Hospital-based	2	1.20 (0.82, 1.75)	97.1%	<0.001	2	0.99 (0.95, 1.02)	94.7%	<0.001
Study areas:								
Europe	5	1.34 (0.94, 1.89)	73.4%	0.005	4	1.01 (0.98, 1.05)	90.1%	<0.001
North America	5	1.13 (1.01, 1.26)	86.7%	<0.001	3	1.03 (0.98, 1.08)	66.4%	0.051
Asia	1	1.01 (0.98, 1.04)	/	/				
Follow-up duration:								
≤10	4	1.11(0.95, 1.31)	70.4%	0.018	1	1.21 (1.06, 1.35)	/	/
>10	7	1.10 (1.03, 1.17)	86.3%	<0.001	4	1.01 (0.99, 1.03)	94.4%	<0.001
Adjusted comorbidity:								
Yes	7	1.10 (1.03, 1.17)	85.3%	<0.001	4	1.04 (0.99, 1.09)	72.6%	0.012
No	4	1.07 (0.90, 1.26)	64.1%	0.039	3	1.00 (0.97, 1.04)	91.2%	<0.001
Adjusted smoking:								
Yes	6	1.21 (1.00, 1.46)	57.0%	0.040	3	1.10 (1.02, 1.19)	53.2%	0.118
No	5	1.06 (1.01, 1.11)	90.2%	<0.001	4	1.00 (0.98, 1.03)	96.5%	<0.001

### PM_2.5_ exposure and the risk of cognitive impairment

**Figure 2:**
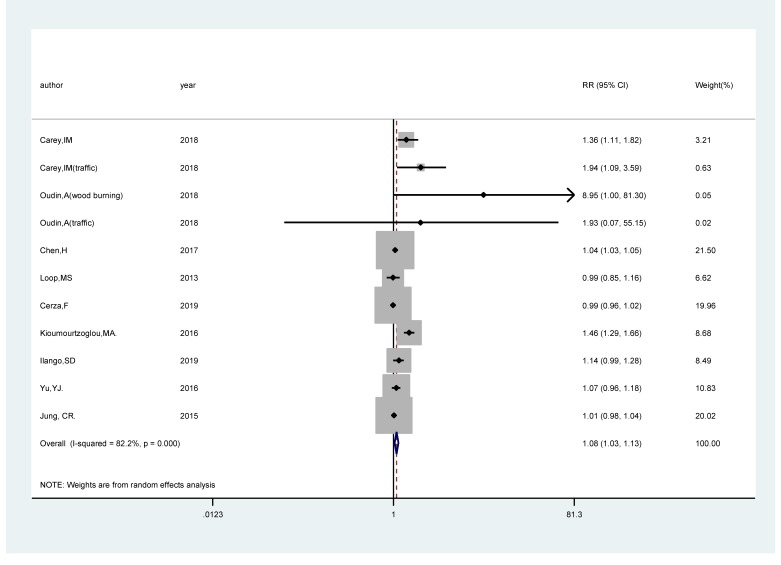
Forest plot for the pooled relative ratio (RR) and 95% confidence interval (CI) of studies on PM_2.5_ exposure (per 5 μg/m^3^ increment) with cognitive impairment. The size of the gray box is positively proportional to the weight assigned to each study, and horizontal lines represent the 95% CI.

Eleven studies [17-25] comprising 519 247 cases among 12 523 553 participants were included. The pooled RR of cognitive impairment per 5 μg/m3 increments in exposure to PM_2.5_ was 1.08 (95% CI = 1.03, 1.13; *I^2^* = 82.2%; *P_heterogeneity_*<0.001; **Figure 2**). A positive significant association (RR*_p_*_er_ *_5 μg/m_^3^ _=_* 1.36; 95% CI = 1.24, 1.50; *I^2^* = 99.7%; *P_heterogeneity_*<0.001; Fig. S1 in the **Online Supplementary Document**) of cognitive impairment with PM_2.5_ exposure was found after sensitivity analysis with the number of participants as the weight.

We carried out subgroup analysis by the source of outcome, study areas, follow-up duration, and adjustment status for comorbidity and smoking. PM_2.5_ revealed a statistically significant positive association with the risk of cognitive impairment in population-based studies (RR*_p_*_er_ *_5 μg/m_^3^ =* 1.05; 95% CI = 1.01, 1.09; *I^2^* = 57.4%; *P_heterogeneity_* = 0.016), whereas no statistically significant association was found in hospital-based studies (RR*_per 5 μg/m_^3^* = 1.20; 95% CI = 0.82,1.75; *I^2^* = 97.1%; *P_heterogeneity_*<0.001). Regarding study areas, the RR*_per 5 μg/m_^3^* were 1.13 (95% CI = 1.01, 1.26; *I^2^* = 86.7%; *P_heterogeneity_*<0.001) for studies conducted in North America, 1.34 (95% CI = 0.94, 1.89; *I^2^* = 73.4%; *P_heterogeneity_* = 0.005) in Europe and 1.10 (95% CI = 0.98, 1.04) in Asia. For follow-up duration, the RR*_per 5 μg/m_^3^* were statistically significant among the studies with following duration >10 years (RR*_p_*_er_ *_5 μg/m_^3^* = 1.10; 95% CI = 1.03, 1.17; *I^2^* = 86.3%; *P_heterogeneity_*<0.001), while the association was not significant among the studies with following duration ≤10 years (RR*_p_*_er_ *_5 μg/m_^3^* = 1.11; 95% CI = 0.95, 1.31; *I^2^* = 70.4%; *P_heterogeneity_* = 0.018). There was a statistically significant positive relationship between PM_2.5_ and the risk of cognitive impairment in studies adjusting for comorbidity (RR*_p_*_er_ *_5 μg/m_^3^* = 1.10; 95% CI = 1.03, 1.17; *I^2^* = 85.3%; *P_heterogeneity_*<0.001)_,_ while no association was found in studies that did not adjust comorbidity (RR*_p_*_er_ *_5 μg/m_^3^* = 1.07; 95% CI = 0.90, 1.26; *I^2^* = 64.1%; *P_heterogeneity_* = 0.039). The combined RR*_per 5 μg/m_^3^* (RR*_per 5 μg/m_^3^* = 1.21; 95% CI = 1.00, 1.46; *I^2^* = 57.0%; *P_heterogeneity_* = 0.040) in smoking-adjusted studies were higher than that (RR*_per 5 μg/m_^3^* = 1.06; 95% CI = 1.01, 1.11; *I^2^* = 90.2%; *P_heterogeneiy_*<0.001) in studies that did not adjust for smoking.

### NO_2_/NO_x_ exposure and the risk of cognitive impairment

**Figure 3:**
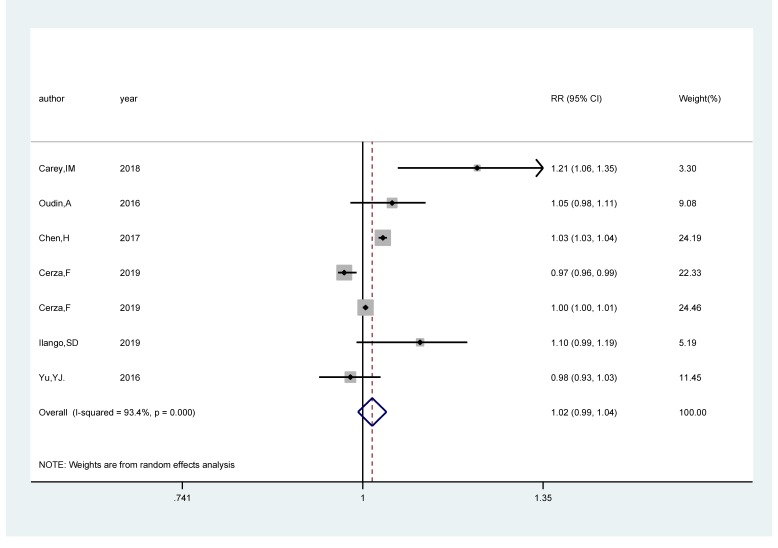
Forest plot for the pooled relative ratio (RR) and 95% confidence interval (CI) of studies on NO_2_/NO_x_ exposure (per 5 ppb increment) with cognitive impairment. The size of the gray box is positively proportional to the weight assigned to each study, and horizontal lines represent the 95% CI.

Seven studies [17,18,20,21,25,26] comprising 285 752 cases among 2 589 907 participants were included. The pooled RR of cognitive impairment per 5 ppb increments in exposure to NO_2_/NO_x_ was 1.02 (95% CI = 0.99, 1.04; *I^2^* = 93.9%; *P_heterogeneity_*<0.001; **Figure 3**). A positive significant association (RR*_p_*_er_ *_5ppb =_* 1.03; 95% CI = 1.02, 1.04; *I^2^* = 96.9%; *P_heterogeneity_*<0.001; Fig.S2 in the **Online Supplementary Document**) of cognitive impairment with NO_2_/NO_x_ exposure was found after sensitivity analysis with the number of participants as the weight.

We carried out subgroup analysis by the source of outcome, study areas, follow-up duration, and adjustment status for comorbidity and smoking. NO_2_/NO_x_ revealed a statistically significant positive association with the risk of cognitive impairment in population-based studies (RR*_p_*_er_ *_5 ppb _=* 1.048; 95% CI = 1.004, 1.094; *I^2^* = 68.5%; *P_heterogeneity_* = 0.013), whereas no association was found in hospital-based studies (RR*_per 5 ppb_* = 0.99; 95% CI = 0.95, 1.02; *I^2^* = 94.7%; *P_heterogeneity_*<0.001). Regarding study areas, we didn’t find relationship in either North America (RR*_p_*_er_ *_5 ppb_* = 1.03; 95% CI = 0.98, 1.08; *I^2^* = 66.4%; *P_heterogeneity_* = 0.051) or Europe (RR*_p_*_er_ *_5 ppb_* = 1.01; 95% CI = 0.98, 1.05; *I^2^* = 90.1%; *P_heterogeneity_*<0.001). There was no relationship between NO_2_/NO_x_ and the risk of cognitive impairment both in studies adjusting for comorbidity (RR*_p_*_er_ *_5 ppb_* _=_ 1.04; 95% CI = 0.99, 1.09; *I^2^* = 72.6%; *P_heterogeneity_* = 0.012) and not adjusting for comorbidity (RR*_p_*_er_ *_5 ppb_* _=_ 1.00; 95% CI = 0.97, 1.04; *I^2^* = 91.2%; *P_heterogeneity_*<0.001). The combined RR*_per 5 ppb_* were statistically significant (RR*_p_*_er_ *_5 ppb_* = 1.10; 95% CI = 1.02, 1.19; *I^2^* = 53.2%; *P_heterogeneity_* = 0.118) in smoking-adjusted studies, but not in studies that did not adjust for smoking (RR*_p_*_er_ *_5 ppb_* _=_ 1.00; 95% CI = 0.98, 1.03; *I^2^* = 96.5%; *P_heterogeneity_*<0.001).

### O_3_ exposure and the risk of cognitive impairment

**Figure 4:**
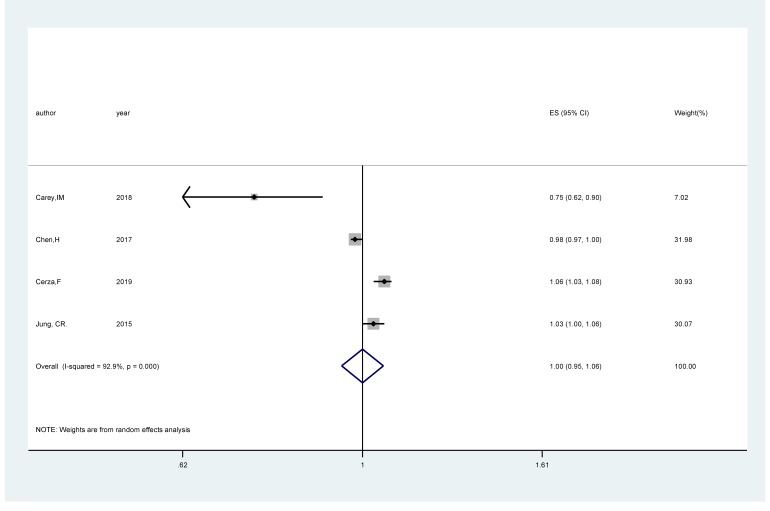
Forest plot for the pooled relative ratio (RR) and 95% confidence interval (CI) of studies on O_3_ exposure (per 5 ppb increment) with cognitive impairment. The size of the gray box is positively proportional to the weight assigned to each study, and horizontal lines represent the 95% CI.

Four studies [17,18,20,22] comprising 282 944 cases among 2 644 151 participants were included. The pooled RR of cognitive impairment per 5 ppb increments in exposure to O_3_ was 1.00 (95% CI = 0.95,1.06; *I^2^* = 92.9%; *P_heterogeneity_*<0.001; **Figure 4**). An inverse significant association (RR*_p_*_er_ *_5ppb _=* 0.98; 95% CI = 0.96, 0.99; *I^2^* = 95.2%; *P_heterogeneity_*<0.001; Fig.S3 in the **Online Supplementary Documen**t) of cognitive impairment with O_3_ exposure was found after sensitivity analysis with the number of participants as the weight.

### Meta-regression

As displayed in **Figure 2**_,_ high heterogeneity (*I^2^* = 82.8%, *P_heterogeneity_* = 0.030) was found in the analysis of PM_2.5_ and the risk of cognitive impairment. Hence, we performed meta-regression with the covariates of the source of outcome (*P* = 0.663), study areas (*P* = 0.403)_,_ follow-up duration (*P* = 0.993), whether adjusted comorbidity (*P* = 0.679) and whether adjusted smoking (*P* = 0.475) to explore potential sources of the heterogeneity. However, no covariates contributed to the heterogeneity of between-study.

In the analysis of NO_2_/NO_x_ and the risk of cognitive impairment, as displayed in **Figure 3** high heterogeneity (*I^2^* = 93.4%, *P_heterogeneity_*<0.001) was found. The meta-regression with the covariates of the source of outcome (*P* = 0.219), study areas (*P* = 0.065)_,_ follow-up duration (*P* = 0.053), whether adjusted comorbidity (*P* = 0.979) and whether adjusted smoking (*P* = 0.540) to explore potential sources of the heterogeneity, and no covariates contributed to the heterogeneity.

### Sensitivity analysis and influence analysis

For the PM_2.5_ exposure and the risk of cognitive impairment, we carried out the leave-one-out sensitivity analysis. After excluding two studies [19,20], *I^2^* decreased from 82.2% to 57.4% (*P* = 0.016), and the result remained significant (RR*_p_*_er_ *_5 μg/m_^3^* = 1.05; 95% CI = 1.01,1.09; Figure S4 in the **Online Supplementary Document**).

In the influence analysis, no individual study had an overmuch impact on the pooled effect for PM_2.5_ and NO_2_/NO_x_ with the risk of cognitive impairment (Figure S5 in the **Online Supplementary Document** and Figure S6 in the **Online Supplementary Document**).

### Publication bias

The visual scrutiny of the funnel plot (Figure S7 in the **Online Supplementary Document** and Figure S8 in the **Online Supplementary Document**) seemed to be asymmetrical for PM_2.5_ and NO_2_/NO_x_, while the Egger’s test displayed no evidence of significant publication bias in the analysis between PM_2.5_ (*P* = 0.155) and NO_2_/NO_x_ (*P* = 0.792) and the risk of cognitive impairment, respectively.

## DISCUSSION

This meta-analysis of longitudinal cohort studies included 10 articles to quantitatively evaluate the association between air pollution exposure and the risk of cognitive impairment. The results demonstrated that exposure to PM_2.5_ was significantly related to an increased risk of cognitive impairment. More specifically, for every 5 μg/m^3^ increase in PM_2.5_ concentration, the risk of cognitive impairment increased by 8%. Nevertheless, there is no statistical association between NO_2_/NO_x_ and O_3_ exposure and the risk of cognitive impairment. And the correlation direction did not change after sensitivity analysis. For PM_2.5_ exposure, subgroup analysis showed significant associations in population-based studies, studies that conducted in North America and studies with follow-up duration >10 years.

Several biological mechanisms have been put forward for the positive relationship of particulate matter (PM) with cognitive impairment. First, exposure to air pollution, particularly PM might cause neuroinflammation, oxidative stress and change brain innate immune responses in early adulthood [36]. At present, inflammation and oxidative stress have been confirmed as basic mechanisms by which air pollution may affect central nervous system disease (CNS) [9]. Second, PM can activate microglia [37], excessive and chronic activation may lead to neurotoxicity [38]. Importantly, microglial activation has been implicated in the progression of diseases such as dementia [39]. Third, the olfactory bulb is another pathway [40] through which PM enters the body, reaching the brain directly and inducing a series of changes such as increased the level of amyloid-β42, hyperphosphorylated τ, and neural degeneration [41,42].

High heterogeneity appeared in this meta-analysis of PM_2.5_ and NO_2_/NO_x_ and the risk of cognitive impairment. To search for potential heterogeneity, we performed the following work. Meta-regression was performed to detect the potential factors that contributed to heterogeneity between studies, however, no factors were found to do with it. We also conducted influence analysis, and the results indicated that no individual study had an excessive impact on the pooled effect of PM_2.5_ and NO_2_/NO_x_ and the risk of cognitive impairment. For PM_2.5_ exposure, the leave-one-out sensitivity analysis indicated that two studies [19,20] affected the heterogeneity and after further excluding two studies, the pooled RR was decreased but not altered substantially. In two studies, the source of outcome was from the hospital-based population, which was likely to not cover all patients and could introduce selection bias.

Our study presents several advantages. First, our meta-analysis included a large number of participants from longitudinal cohort studies, providing high statistical power and making it more likely to obtain a reasonable conclusion. Second, the pooled RR of this meta-analysis was based on data on dose-response relationships in the original studies, thus the existence of causation was further supported. Furthermore, to minimize between-study variation, we normalized the exposure levels of PM_2.5_, NO_2_/NO_x_ and O_3_ across studies into uniform units, and the pooled RR was standardized per 5μg/m^3^ increments for PM_2.5_ and 5 ppb for NO_2_/NO_x_ and O_3_. Third, original studies were all fully taken into account potential confounders such as age. Moreover, sex, comorbidities, and smoking were adjusted for in most studies. As well, the quality assessment score of each study was higher than 7, demonstrating that the quality of the included articles was generally good.

Nevertheless, several limitations of our meta-analysis should be recognized. First, although as much as possible potential confounders were adjusted for in most studies, several studies still did not adjust for potential confounders, such as comorbidities. Comorbidities include heart disease, stroke, diabetes, hypertension and so on, which may elevate cognitive impairment risk [43-45]. In the analysis of PM_2.5_ and cognitive impairment, a significant association was found in studies adjusting for comorbidity, but no association was found in studies that did not adjust for comorbidity, therefore the combined result was underestimated. Second, varied exposure assessment methods differed in its ability to provide estimates of individual exposure levels, which might increase the instability of the results to some extent. Third, the definition of cognitive impairment was inconsistent, resulting in misclassification bias.

## CONCLUSIONS

In summary, the results of this meta-analysis demonstrated that exposure to PM_2.5_ was associated with an increase in cognitive impairment risk. The results may have substantial public health significance for the prevention of cognitive impairment through air pollution interventions.

## Additional material

Online Supplementary Document
