# Supplementary material for: Exposure to air pollution and cognitive impairment risk: a meta-analysis of longitudinal cohort studies with dose-response analysis
Source: J Glob Health. 2020 Apr 19;10(1):010417. doi: 10.7189/jogh.10.010417 (PMC7182361; doi:10.7189/jogh.10.010417)
Supplement: Online Supplementary Document [file jogh-10-010417-s001.pdf]

Table S1 The search strategy of air pollution and cognitive impairment in PubMed

Step	Search term (the number of articles)
#1	air pollution (74146)
#2	particulate matter (68978)
#3	carbon monoxide (35041)
#4	nitrogen dioxide (16102)
#5	nitrogen oxide (119241)
#6	sulfur dioxide (8985)
#7	ozone (23094)
#8	#1 OR #2 OR #3 OR #4 OR #5 OR #6 OR #7 (285015)
#9	cognitive impairment (97224)
#10	dementia (201291)
#11	#9 OR #10 (266902)
#12	#8 AND #11(1085)

Table S2 Detailed list of the number of exclude full-text reviewed articles

Step	The number of articles before exclusion	Excluding reasons	The number of articles after exclusion	References number of excluded articles
1	41	Exposure of interest was not air pollution	37	1-4
2	37	Outcome of interest was not cognitive impairment	34	5-7
3	34	Can not be converted into *RR* with per increment	17	8-24
4	17	Duplicate from the same population	13	25-28
5	13	Not cohort studies	10	29-31

References

1. Chen, R., *Association of environmental tobacco smoke with dementia and Alzheimer's disease among never smokers.* Alzheimers Dement, 2012. **8**(6): p. 590-5.
2. Chen, R., et al., *Association between environmental tobacco smoke exposure and dementia syndromes.* Occup Environ Med, 2013. **70**(1): p. 63-9.
3. Krishnamoorthy, Y., et al., *Association between Indoor Air Pollution and Cognitive Impairment among Adults in Rural Puducherry, South India.* J Neurosci Rural Pract, 2018. **9**(4): p. 529-534.
4. Malmqvist, E., et al., *Estimated health benefits of exhaust free transport in the city of Malmo, Southern Sweden.* Environ Int, 2018. **118**: p. 78-85.
5. Li, R.L., et al., *Influence of PM2.5 Exposure Level on the Association between Alzheimer's Disease and Allergic Rhinitis: A National Population-Based Cohort Study.* Int J Environ Res Public Health, 2019. **16**(18).
6. Polito, C., et al., *Indoor Air Pollution May Be Associated with Cognitive Impairment in Chronic Obstructive Pulmonary Disease.* American Journal of Respiratory and Critical Care Medicine, 2018. **197**: p. 2.
7. Porta, D., et al., *Air Pollution and Cognitive Development at Age 7 in a Prospective Italian Birth Cohort.* Epidemiology, 2016. **27**(2): p. 228-36.
8. Cacciottolo, M., et al., *Particulate air pollutants, APOE alleles and their contributions to cognitive impairment in older women and to amyloidogenesis in experimental models.* Transl Psychiatry, 2017. **7**(1): p. e1022.
9. Chang, K.H., et al., *Increased risk of dementia in patients exposed to nitrogen dioxide and carbon monoxide: a population-based retrospective cohort study.* PLoS One, 2014. **9**(8): p. e103078.
10. Cleary, E.G., et al., *Association of Low-Level Ozone with Cognitive Decline in Older Adults.* J Alzheimers Dis, 2018. **61**(1): p. 67-78.
11. Cullen, B., et al., *Cross-sectional and longitudinal analyses of outdoor air pollution exposure

and cognitive function in UK Biobank. Scientific Reports, 2018. **8**: p. 14.

12. Gatto, N.M., et al., *Components of air pollution and cognitive function in middle-aged and older adults in Los Angeles.* Neurotoxicology, 2014. **40**: p. 1-7.
13. Huls, A., et al., *The role of air pollution and lung function in cognitive impairment.* Eur Respir J, 2018. **51**(2).
14. Lee, H., et al., *Exposure to ambient fine particles and neuropsychiatric symptoms in cognitive disorder: A repeated measure analysis from the CREDOS (Clinical Research Center for Dementia of South Korea) study.* Sci Total Environ, 2019. **668**: p. 411-418.
15. Power, M.C., et al., *Traffic-related air pollution and cognitive function in a cohort of older men.* Environ Health Perspect, 2011. **119**(5): p. 682-7.
16. Qiu, H., et al., *Attributable risk of hospital admissions for overall and specific mental disorders due to particulate matter pollution: A time-series study in Chengdu, China.* Environmental Research, 2019. **170**: p. 230-237.
17. Ranft, U., et al., *Long-Term Exposure to Traffic-Related Air Pollution and Mild Cognitive Impairment in the Elderly.* Epidemiology, 2009. **20**(6): p. S22-S22.
18. Saenz, J.L., R. Wong, and J.A. Ailshire, *Indoor air pollution and cognitive function among older Mexican adults.* Journal of Epidemiology and Community Health, 2018. **72**(1): p. 21-26.
19. Salinas-Rodriguez, A., et al., *Exposure to ambient PM2.5 concentrations and cognitive function among older Mexican adults.* Environment International, 2018. **117**: p. 1-9.
20. Tallon, L.A., et al., *Cognitive impacts of ambient air pollution in the National Social Health and Aging Project (NSHAP) cohort.* Environment International, 2017. **104**: p. 102-109.
21. Tzivian, L., et al., *Long-term air pollution and traffic noise exposures and cognitive function:A cross-sectional analysis of the Heinz Nixdorf Recall study.* Journal of Toxicology and Environmental Health-Part a-Current Issues, 2016. **79**(22-23): p. 1057-1069.
22. Tzivian, L., et al., *Associations of long-term exposure to air pollution and road traffic noise with cognitive function-An analysis of effect measure modification.* Environment International, 2017. **103**: p. 30-38.
23. Wu, Y.C., et al., *Association between air pollutants and dementia risk in the elderly.* Alzheimers Dement (Amst), 2015. **1**(2): p. 220-8.
24. Wurth, R., et al., *Fine Particle Sources and Cognitive Function in An Older Puerto Rican Cohort in Greater Boston.* Environ Epidemiol, 2018. **2**(3).
25. Andersson, J., et al., *Road traffic noise, air pollution, and risk of dementia - results from the Betula project.* Environ Res, 2018. **166**: p. 334-339.
26. Chen, H., et al., *Living near major roads and the incidence of dementia, Parkinson's disease, and multiple sclerosis: a population-based cohort study.* Lancet, 2017. **389**(10070): p. 718-726.
27. Lee, M., et al., *Long-term effect of fine particulate matter on hospitalization with dementia.* Environ Pollut, 2019. **254**(Pt A): p. 112926.
28. Oudin, A., et al., *Traffic-Related Air Pollution as a Risk Factor for Dementia: No Clear Modifying Effects of APOEvarepsilon4 in the Betula Cohort.* Radiol Med, 2019.
29. Culqui, D.R., et al., *Association between environmental factors and emergency hospital admissions due to Alzheimer's disease in Madrid.* Sci Total Environ, 2017. **592**: p. 451-457.
30. Linares, C., et al., *Short-term association between environmental factors and hospital admissions due to dementia in Madrid.* Environ Res, 2017. **152**: p. 214-220.

31. Tzivian, L., et al., *Long-Term Air Pollution and Traffic Noise Exposures and Mild Cognitive Impairment in Older Adults: A Cross-Sectional Analysis of the Heinz Nixdorf Recall Study.* Environmental Health Perspectives, 2016. **124**(9): p. 1361-1368.

Table S3 Quality assessment of included cohort studies

First author, (year)	Representativeness of the exposed cohort	Selection of the unexposed cohort	Ascertainment of exposure	Outcome of interest not present at start of study	Control for important factor or additional factor[2]	Outcome assessment	Follow-up long enough for outcomes to occur[3]	Adequacy of follow-up of cohorts[4]	Overall quality score
Carey,IM. (2018)	*		*	*	**	*	*	*	8
Oudin,A. (2016)	*		*	*	**	*	*	*	8
Oudin,A. (2018)	*		*	*	**	*	*	*	8
Chen,H. (2017)	*		*	*	**	*	*	*	8
Cerza,F (2019)	*		*	*	**	*	*	*	8
Kioumourtzoglou,MA. (2016)	*		*		**	*	*	*	7
Ilango,SD (2019)	*		*	*	* *	*	*	*	8
Yu,YJ. (2016)	*		*	*	* *	*	*	*	8
Jung, CR. (2015)	*		*	*	**	*	*	*	8
Loop,MS.	*		*	*	**	*	*	*	8

(2013)

[1] A study could be awarded a maximum of one star for each item except for the item Control for important factor or additional factor. A maximum of 2 stars could be awarded for this item. Studies that controlled for age or gender received one star, whereas studies that controlled for other important confounders such as chronic health conditions received an additional star.

[2] A cohort study with a follow-up time >3 year was assigned one star.

[3] A cohort study with a follow-up rate >70% was assigned one star.

[4] One star represents a score of 1, and a study can be awarded a maximum score of 9 (9 stars) in total.

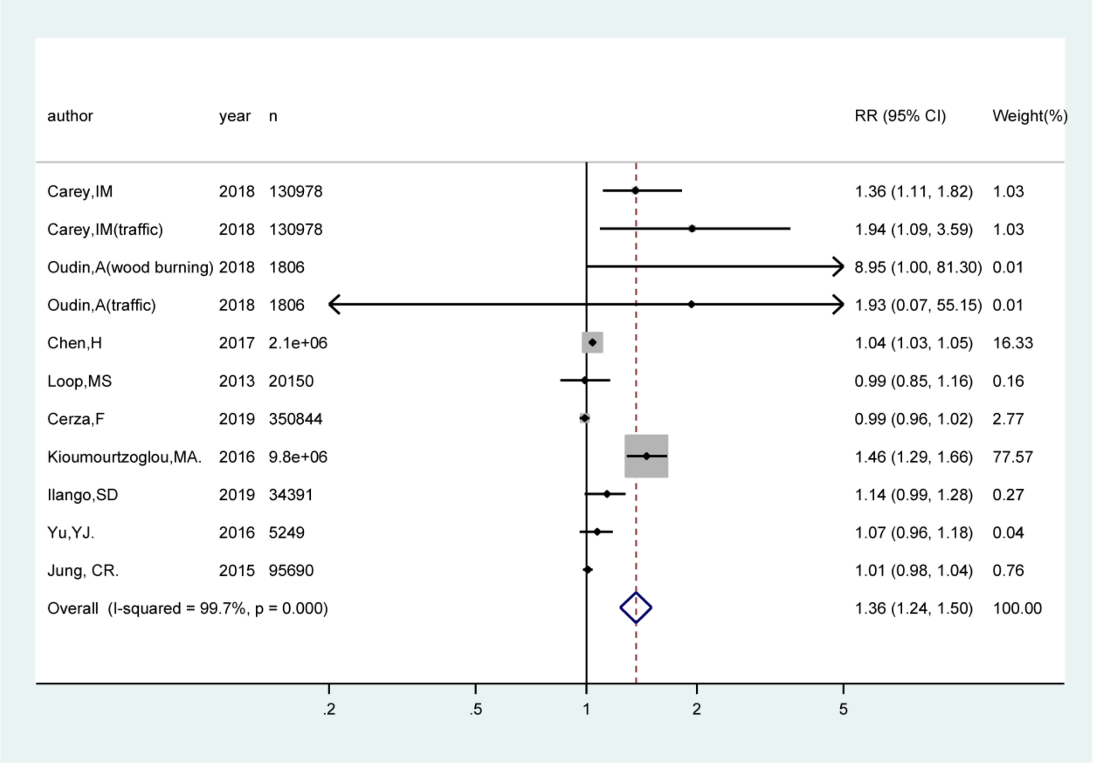

Figure.S1 Forest plot for the pooled *RR* and 95 %*CI* of studies on $PM_{2.5}$ exposure (per 5 μg/m^3 increment) with cognitive impairment. The size of the grey box is positively proportional to the weight assigned to each study, and horizontal lines represent the 95%*CI*

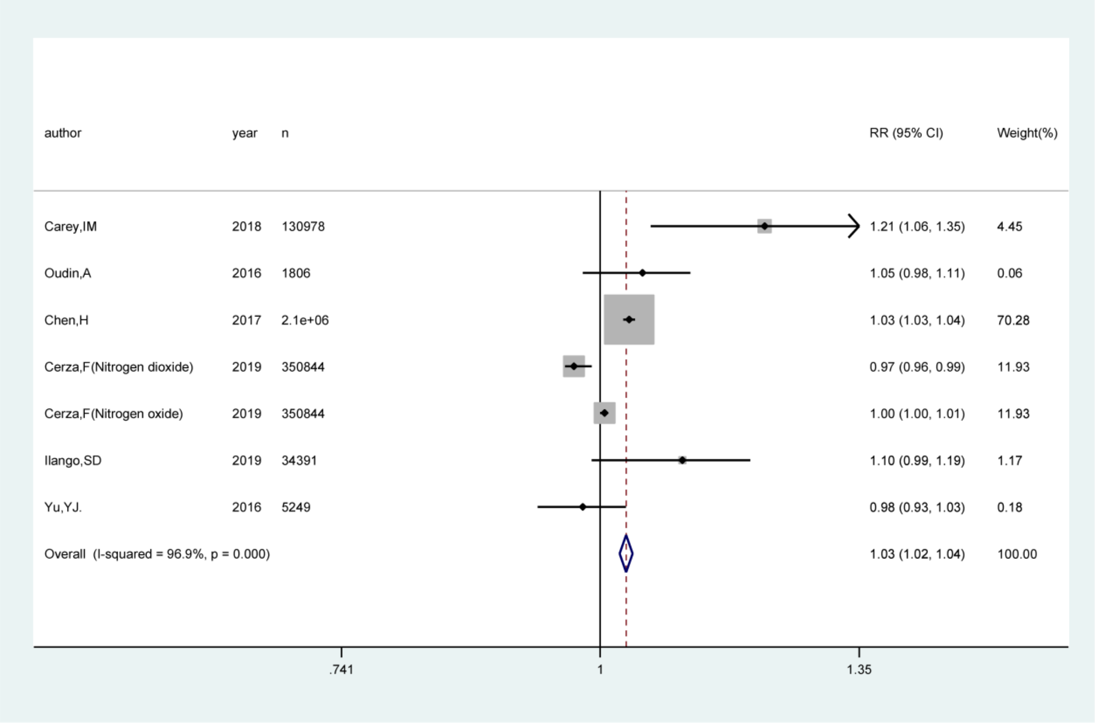

Figure.S2 Forest plot for the pooled *RR* and 95 %*CI* of studies on NO_2/NO_x exposure (per 5 ppb increment) with cognitive impairment. The size of the grey box is positively proportional to the weight assigned to each study, and horizontal lines represent the 95%*CI*

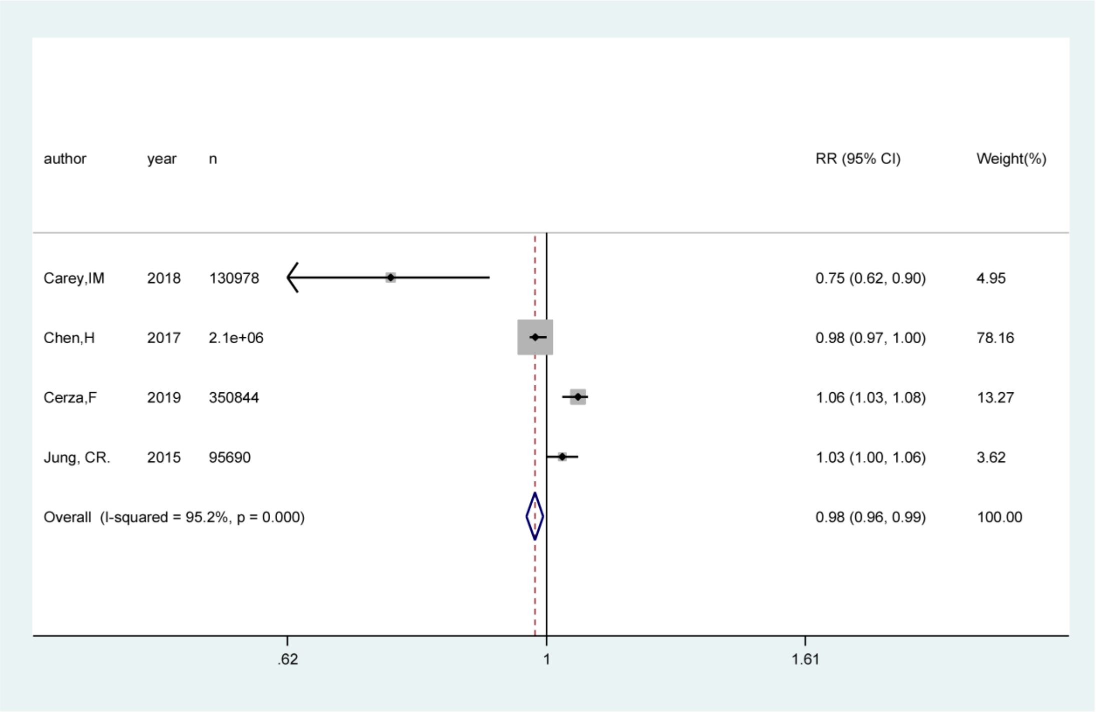

Figure.S3 Forest plot for the pooled *RR* and 95 %*CI* of studies on O_3 exposure (per 5 ppb increment) with cognitive impairment. The size of the grey box is positively proportional to the weight assigned to each study, and horizontal lines represent the 95%*CI*

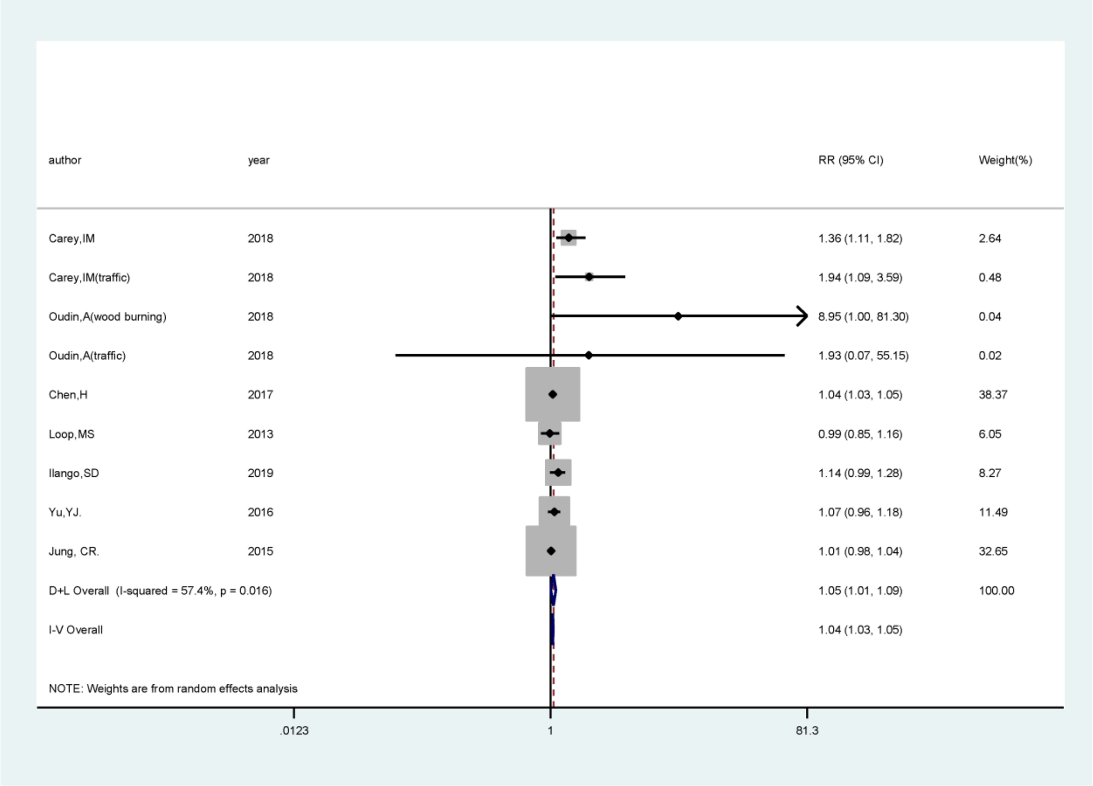

Figure.S4 Forest plot for the pooled *RR* and 95 %*CI* of studies on $PM_{2.5}$ exposure (per 5 μg/m^3 increment) with cognitive impairment (excluding two studies that contributed to the between-study heterogeneity). The size of the grey box is positively proportional to the weight assigned to each study, and horizontal lines represent the 95%*CI*

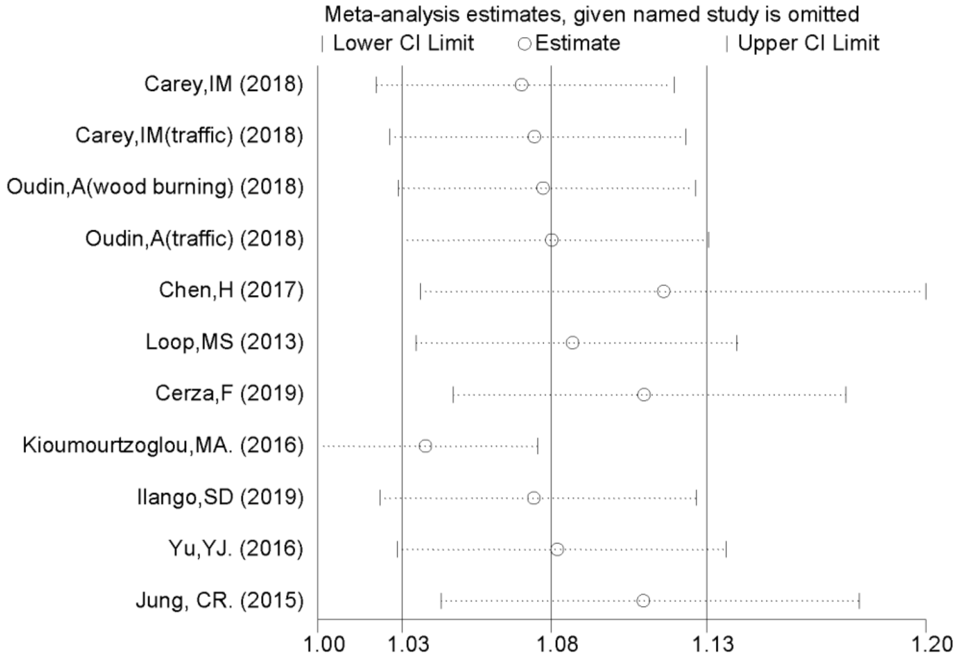

Figure.S5 Influence analysis of 11 studies on $PM_{2.5}$ and the risk of cognitive impairment

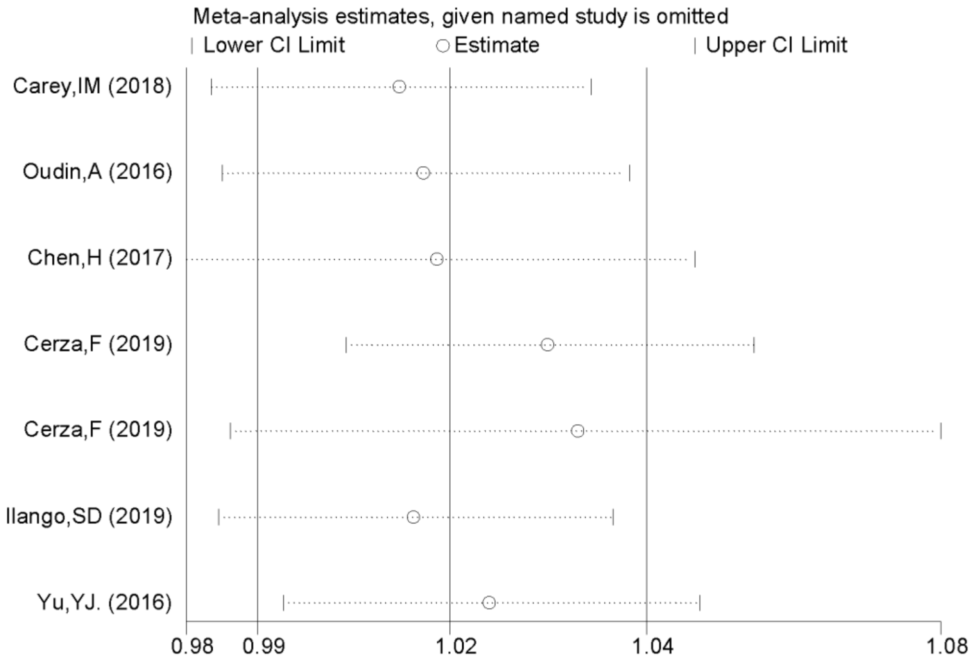

Figure.S6 Influence analysis of 7 studies on NO_2/NO_x and the risk of cognitive impairment

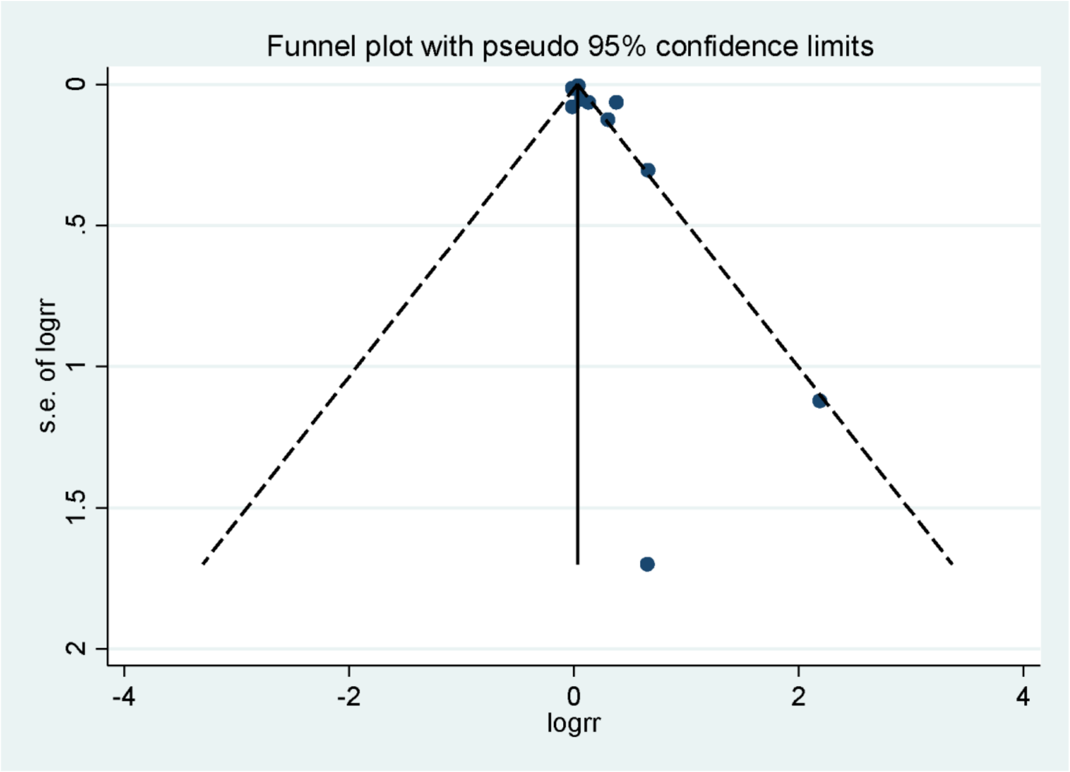

Fig.S7 Funnel plot of the relative risk of $PM_{2.5}$ exposure and cognitive impairment.

Each dot represents a different study

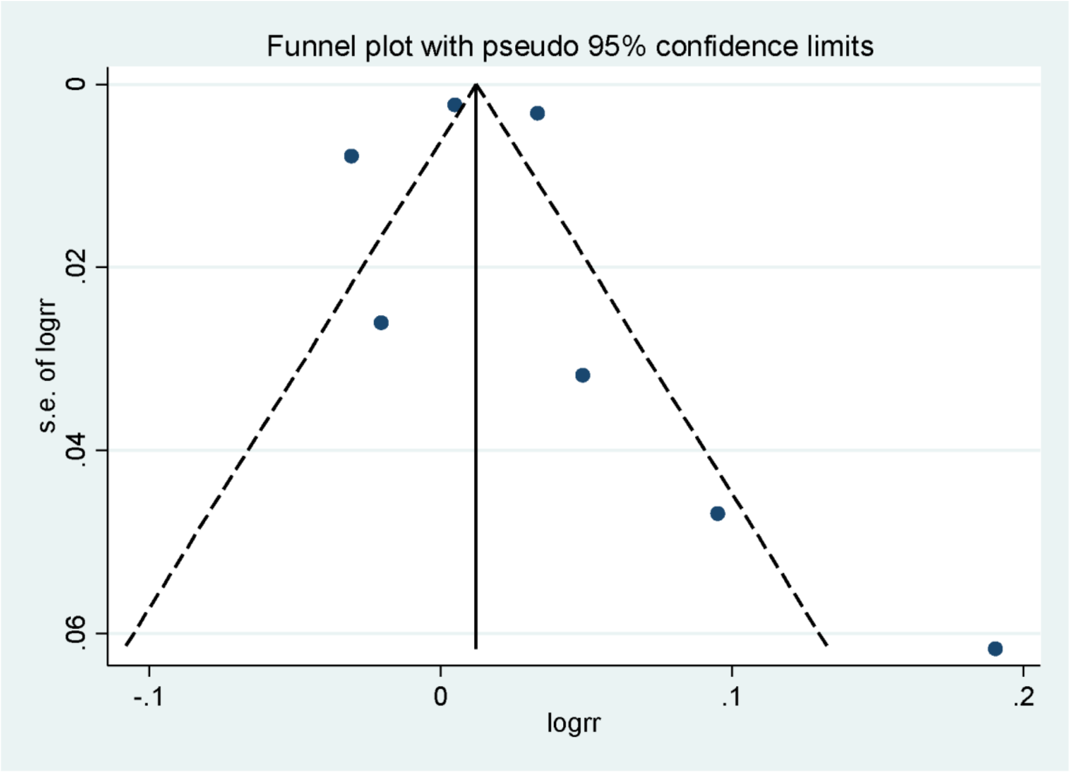

Fig.S8 Funnel plot of the relative risk of NO_2/NO_x exposure and cognitive impairment.

Each dot represents a different study
